# Bio-Based Aqueous Dispersions Based on Unsaturated PLA Polymers for Barrier Packaging Applications

**DOI:** 10.3390/polym17182467

**Published:** 2025-09-12

**Authors:** Roosa Hämäläinen, Pauliina Kivinen, Rajesh Koppolu, Eetu Nissinen, Adina Anghelescu-Hakala

**Affiliations:** VTT Technical Research Centre of Finland Ltd., P.O. Box 1000, FI-02044 Espoo, Finland; roosa.hamalainen@vtt.fi (R.H.); pauliina.kivinen@vtt.fi (P.K.); rajesh.koppolu@vtt.fi (R.K.); eetu.nissinen@vtt.fi (E.N.)

**Keywords:** poly(lactic acid) copolymer, bio-based, polycondensation, dispersions, coatings

## Abstract

The growing demand for sustainable packaging materials highlights the need for bio-based alternatives to fossil-derived polymers, particularly in barrier applications where reduced environmental impact and recyclability are critical. Poly(lactic acid) is a promising candidate due to its renewable origin and biodegradability, yet its application in aqueous dispersion coatings remains underdeveloped. In this study, copolymers were synthesized from L-(+)-lactic acid, itaconic acid, and 1,4-/2,3-butanediol via polycondensation, and a solvent-free thermomechanical method was used to prepare aqueous dispersions from the produced copolymers. The main objective of this study was to identify an optimal composition for the copolymer and dispersion to achieve small and uniformly sized dispersion particles while also assessing the scalability of the process from laboratory to pilot production. The smallest dispersion particles and most uniform size distribution were achieved with a copolymer that had an M_n_ close to the average (10,180 g mol^−1^) and a low T_g_ (−1.4 °C). The grade and dosage of the dispersion stabilizer significantly influenced the particle size and particle size distribution. The process scale-up, including polymer production at pilot scale and dispersion preparation at bench scale, was successfully demonstrated. The water vapor barrier properties of the coated dispersions were promising (<10 g/m^2^ at 23 °C/50% RH), supporting the potential of aqueous PLA-based dispersions as sustainable barrier coatings.

## 1. Introduction

Since the environmental issues related to fossil fuels and the finiteness of their resources were discovered, decades have been dedicated to finding strategies to replace the fuels in most of their applications [1,2]. In the packaging industry, this means finding renewable alternatives for petroleum-based polymers, which are utilized in barrier coatings of packaging applications [3]. Barrier dispersion coatings provide a protective barrier layer against grease, oil, water, water vapor, gases, and other impurities and thus impose several performance requirements on the coating material [4].

Current water-based dispersions (latexes) used as coatings, binders, and adhesives are mainly based on polymers derived from crude oil, and only a few are compostable and even fewer biodegradable. The global synthetic latex polymers market reflects the continued reliance on these materials, with a valuation of USD 40.6 billion in 2024, projected to reach USD 53.2 billion by 2029, at a compound annual growth rate (CAGR) of 5.5% [5]. Latexes produced from fossil-based polymers such as styrene-butadiene rubber, acrylic polymers, and ethylene vinyl acetate (EVA) are widely used due to their effective barrier properties. Their environmental impacts are high, and recycling and upcycling these polymers are challenging. Recent developments aim to replace conventional polymers derived from non-renewable feedstocks with novel bio-based polymers that are more sustainable, safer, and equal or superior in performance compared to the current materials.

Being completely bio-based and to some extent biodegradable, poly(lactic acid) (PLA), a thermoplastic aliphatic polyester [6], is considered a potential candidate for substituting the conventional petroleum-based plastics, making it one of the most studied biopolyesters [7]. Common challenges hindering the utilization of PLA and other biopolymers in coating applications are their brittleness, toughness, hydrophilicity, sealability, crystallization behavior, and melting instability [8,9]. These limitations can be mitigated through blending the polymer with filler materials or plasticizers or through surface modification [10]. Other biopolymer classes—including proteins, lipids, and polysaccharides—are also potential candidates for sustainable food packaging, yet their widespread use is restricted by brittleness, poor water resistance, or high processing costs, even when nanocomposite fillers or plasticizers are incorporated [11,12,13]. In this context, the present work introduces materials with tunable barrier and mechanical performance while overcoming the hydrophilicity and brittleness commonly associated with biopolymer-based films. Some examples of promising bio-based coating solutions worth mentioning include high-solid PLA dispersions stabilized with PEG-PLA-PEG block copolymers, which enable room-temperature processing, long-term stability, and recyclability while providing effective hydrophobic coatings on paper [14]. Suberin-based aqueous dispersions derived from biomass waste offer fully bio-based, solvent-free coatings with excellent water vapor and heptane vapor barrier properties as well as grease resistance and oil penetration properties, eliminating the need for synthetic additives [15]. Further refinement of this system incorporates amphiphilic cellulose nanofibers as stabilizers and reinforcing agents, resulting in multifunctional films that provide UV protection, antimicrobial activity, and moisture barrier performance suitable for active food packaging applications [16]. Hemicellulose-derived coatings valorize agricultural waste streams to produce hydrophobic, moisture-resistant films, supporting circular economy principles with a low environmental footprint [17]. While these advances showcase impressive functionality and sustainability, challenges remain in scalability, formulation complexity, and balancing mechanical properties with biodegradability.

In packaging, the barrier coating is often applied as a solution or a dispersion [18]. Many of the common methods utilize extensive amounts of organic solvents in the dissolution process, which, from an environmental and economic point of view, is not feasible [14,19,20,21,22,23]. Aiming for a solvent-free process has led to the discovery of thermomechanical dispersion preparation methods, which are applicable for aqueous dispersions of PLA and other biodegradable polymers [8,21,24,25].

In patent literature, several patents related to aqueous dispersions of PLA have been reported. In these, the main dispersion preparation method is solvent-based [26,27,28], and polyvinyl alcohol is a common alternative for the surfactant [27,28,29]. In the patent of Tomomitsu, an aqueous dispersion is prepared from a poly(lactic acid)-based resin, an emulsifier, a plasticizer, and water at a temperature ≥100 °C [30]. Similarly, in the patent of Moireau and Cretu, a dispersion is prepared from either polylactides or polyhydroxyalkanoates, a surfactant, a plasticizer, and water at an elevated temperature and under stirring [31].

In the previous studies by our group, low molecular weight PLA copolymers (PLAX polymers) were prepared from oil-based D,L-lactic acid, 1,4-butanediol, and itaconic acid via direct polycondensation, and the copolymers were dispersed in water with a thermomechanical method. The double bond functionalities of the dispersed polymer were then crosslinked to enhance the thermal and mechanical properties of the polymers. The coated PLA dispersions contained additional additives, including thermal or a combination of thermal and UV crosslinking agents, to enhance the crosslinking during the drying step of the coated dispersions. The barrier properties of the crosslinked dispersions were also evaluated with pilot-scale coating studies, and the results indicated that the materials have potential in the packaging applications of dry and fatty goods [8,25]. More recently, our group investigated the application for barrier packaging of upscaled multilayer dispersion coatings consisting of PLAX coating and a hybrid nanomaterial lacquer (bioORMOCER^®^) [32].

The aim of this study was to focus on the development of optimal dispersion formulation on a laboratory scale, including the production of PLA copolymers with different compositions. The scale-up of both processes was also demonstrated. The effects of the grade and dosage of the dispersion stabilizer and homogenizer post-treatment were elucidated, and the feasibility of PLA dispersions as barrier coatings for paper and paperboard was demonstrated with a roll-to-roll semi-pilot SUTCO (surface treatment concept) coater.

In the present work, the PLAX polymers are prepared from bio-based L-(+)-lactic acid, and the PLA dispersions do not contain any additives to accelerate the crosslinking reactions, marking a novel approach compared to our earlier studies. This work contributes to paving the way towards bio-based sustainable packaging solutions, offering benefits to the industry by lowering the environmental impact and increasing the recycling rate with existing recycling loops of fiber-based packaging materials.

## 2. Materials and Methods

### 2.1. Materials

PLA copolymers (PLAX polymers) were produced from 80% L-(+) lactic acid solution (Orikem; Kangasala, Finland) or 90% L-(+) lactic acid solution (Thermo Scientific; Leicestershire, UK), 1,4-butanediol (Sigma-Aldrich; St. Louis, MO, USA) or 2,3-butanediol (Sigma-Aldrich), itaconic acid (Acros Organics, Novasol Chemicals; Fair Lawn, NJ, USA), and Tin(II) 2-ethylhexanoate (Sigma-Aldrich; St. Louis, MO, USA). Commercial poly(vinyl alcohols) (PVA, grades Poval 5-74, 26-80, 30-75, and 40-88, Kuraray; Tokyo, Japan) were used as dispersion stabilizers for the preparation of PLAX dispersions. The physical properties of the tested grades are shown in Table S1. Upscaled polymer dispersions were coated on two commercial paper grades: UPM AsendoTM and UPM SolideTM Lucent (supplied by UPM).

### 2.2. Preparation of PLAX Polymers

The PLAX polymers (referred to as P.1, P.2, etc.) were synthesized in a 2 L batch reactor from Juchheim Laborgeräte GmbH; Bernkastel-Kues, Germany, which was equipped with a nitrogen inlet, vacuum distillation unit, oil heater, temperature controller, and data collection system for recording the temperature-pressure profiles. The polymerization process was scaled up to a 10 L high-speed batch reactor from Gebrüder Lödige Maschinenbau GmbH, Paderborn, Germany, which had the same functions as the 2 L reactor.

In a typical polymer synthesis, the reagents and catalysts were added to the reactor at room temperature under a nitrogen flow. The temperature was gradually increased to 180 °C over a few hours, and the pressure was reduced from full vacuum to 30 mbar, the final value depending on the batch. The reaction time was 24 h after reaching the final vacuum level. The polymer was cooled down under a nitrogen flow and collected.

### 2.3. Preparation of Aqueous PLAX Dispersions

PLAX dispersions (referred to as D.1, D.2, etc.) were prepared on a laboratory scale with a thermomechanical method using a 1 L glass reactor [8,25]. In a typical procedure, 30 g of PLAX polymer was melted in the reactor at 100–130 °C and when the sample reached a low viscosity, the mixing (60 rpm) was initiated. Next, the temperature was lowered to the desired temperature of 80–125 °C, where the viscosity of molten polymer reached a suitable level for dispersion preparation. The dispersion was stabilized with preheated (70 °C) 7 wt% aqueous PVA solution, which was added in seven portions with the addition intervals varying between 5 and 15 min. The stabilizer addition phase was followed by intensive mixing at 200 rpm, cooling down to 35–40 °C, dilution to the desired solid content (32–33 wt%), and filtration through a fine metallic mesh sieve. As a post-treatment, the dispersions were homogenized with an Ultra-Turrax^®^ T18 digital homogenizer from IKA, Staufen, Germany, which was applied for 15 min at the speed of 10,000–12,000 rpm.

### 2.4. Scale-Up of PLAX Dispersion Preparation

The dispersion process was scaled up to a 2 L batch reactor (Juchheim Laborgeräte GmbH) in a pressurized atmosphere. The reactor was equipped with a heated feeding system, temperature control, a nitrogen inlet, and a data collection system for recording the temperature-pressure profiles.

Firstly, the PLAX polymer was melted at 150 °C in the reactor and mixed at 80 rpm for 20 min. Once fully molten, reactor pressure was increased to 5–6 barg with nitrogen. The feeding line was preheated to 150 °C before the 7 wt% PVA solution was gradually added to the reactor through the feeding system over one hour. The dispersion was cooled down with the mixing speed increased to 150 rpm for 10 min. When the dispersion temperature had reached 40–55 °C, the mixing speed was lowered back to 80 rpm, and the overpressure was gently released for 10 min. The dispersion was diluted with water to the desired solid content and filtered.

### 2.5. Coating of PLAX Dispersions

Dispersions D.13 and D.14 were coated on paper substrates using a semi-pilot roll-to-roll SUTCO (Surface Treatment and Coating Line) coating line. Coatings were applied using the rod coating method (rod for 28 µm wet deposit). The coating speed was 4 m/min. Coatings were dried using 3 infrared and 5 air dryers. Coated samples were conditioned at laboratory conditions of 23 °C and 50% relative humidity before characterization.

### 2.6. Characterizations

#### 2.6.1. Size Exclusion Chromatography

Molecular weights and molecular distributions of the produced copolymer were determined by size exclusion chromatography (SEC) using 1,1,1,3,3,3-hexafluoro-2-propanol (HFIP, 99.9%, Fluorochem, Glossop, UK) as eluent with 5 mM sodium trifluoroacetate (98%, Sigma-Aldrich). The PLAX samples were dissolved overnight at a concentration of 4 mg/mL and filtered (0.45 µm) before the measurement. The measurements were performed at 40 °C, and the eluent delivery rate was 0.5 mL/min. The system was equipped with Waters Styragel columns and a Waters 2410 refractive index detector. The results were calibrated against poly(methyl methacrylate) standards from Agilent Technologies Deutschland GmbH (Waldbronn, Germany).

#### 2.6.2. Differential Scanning Calorimetry

The samples were analyzed with a DSC2 differential scanning calorimeter (Mettler Toledo; Columbus, OH, USA) combined with a TC100MT intra-cooler (Huber; Raleigh, NC, USA) to determine the melting temperature (Tm) and glass transition temperature (Tg). Samples of 3–6 mg were weighed into 40 µL aluminum crucibles, lids were pricked, and crucibles were closed with cold pressing. The samples were heated twice with a 10 °C/min heating rate. During the first scan, the samples were cooled down to −50 °C and heated to 75 °C. In the second scan, the samples were cooled down to −50 °C and heated to 250 °C. The results were processed with Mettler Toledo STARe software (version 13.0).

#### 2.6.3. NMR Spectroscopy

^1^H NMR spectra were recorded on a Bruker Avance III (500 MHz) spectrometer (Karlsruhe, Germany) in CDCl_3_. The number of scans was 16, and the delay time was 25 s.

#### 2.6.4. Particle Size Analysis

Particle size distributions of the dispersion samples were analyzed using a Mastersizer 3000 particle size analyzer combined with a Hydro LV 0.6 L wet dispersant unit, both from Malvern (Malvern, UK). The measurements were performed within one day of dispersion preparation, and prior to analysis, the samples were diluted with distilled water at a 1:1 ratio. The sample was added dropwise into the unit chamber until an obscuration level of 12–13% was reached. The following settings were used in the measurements: Mie scattering as the scattering model, rubber latex particle (refractive index 1.525) as the sample material, and water (refractive index 1.330) as the dispersant. Three samples were analyzed from each dispersion, and each sample was measured at least five times. The cumulative particle sizes were reported as averages from these measurements.

#### 2.6.5. Barrier Properties

Water vapor transmission rate (WVTR) was measured using the gravimetric method by applying the standard ISO 2528:2017 [33]. The samples were measured at 23 °C, 50% RH. The samples were conditioned in the test conditions for at least 12 h before the measurement. EZ-Cup Vapometer cups (Thwing-Albert Instrument Company, West Berlin, NJ, USA) were filled with calcium chloride powder (Thermo Scientific Chemicals; Heysham, UK), and samples were placed on top of the cups, barrier-coated side upwards, between two neoprene gaskets. The test setups were kept in the conditioning chamber (CTC256, Memmert GmbH; Schwabach, Germany) in predetermined conditions for 24 h. The WVTR values (g/(m^2^∙day)) were calculated by comparing the mass after 24 h with the initial mass. Three parallel tests were executed.

The methods for characterization of the remaining dispersion properties and for coat weight and thickness are presented in the Method Descriptions S1 and S2 of the Supporting Information.

## 3. Results and Discussion

### 3.1. Synthesis of PLA Copolymers and Scale-Up Studies

**Figure 1:**
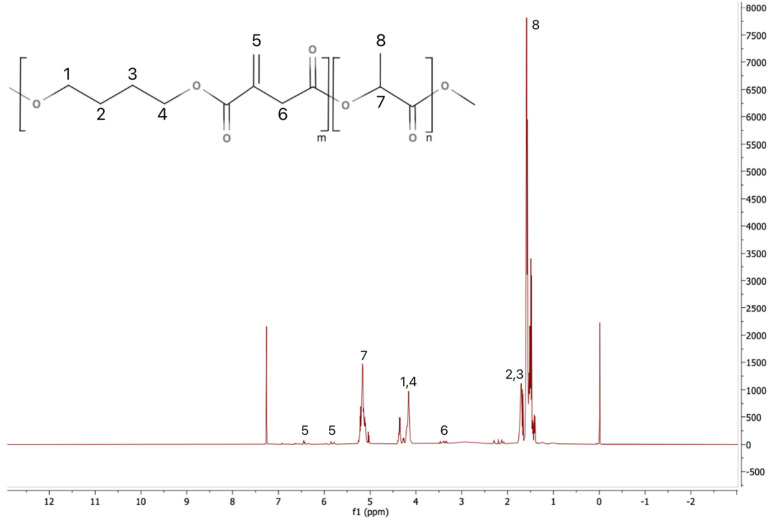
An assigned ^1^H NMR spectrum of a PLAX copolymer (P.4).

The statistical PLA copolymers, referred to as PLAX, were synthesized via polycondensation from bio-based L(+)-lactic acid, itaconic acid, butanediol, and Sn(II)Oct as a catalyst. The polymerizations occur between the hydroxyl group of butanediol and the carboxylic groups of lactic acid and itaconic acid, and water is distilled off from the reaction mixture. The polymerization kinetics can be altered by the presence of itaconic acid due to its reactivity and steric effects. For instance, the presence of unsaturated double bonds in the itaconic acid structure can induce side reactions such as radical crosslinking or Michael addition, providing branching or crosslinking potential. The chemical composition was determined by ^1^H NMR as reported in previous studies [8,25]. The ^1^H NMR spectrum of one representative PLAX polymer is shown in Figure 1. The spectra of the other prepared polymers are very similar and are also consistent with those obtained in our previous studies [8,25].

Table 1 shows the monomer composition in the feed and the monomer unit composition in the copolymer. The molar percentages were determined as previously described [8,25], based on the integration of characteristic NMR signals (LA, IA, BD). The monomer composition of the copolymer was similar to that of the feed, apart from itaconic acid, which was present in a lower proportion than expected based on the feed ratio. This has also been seen in previous studies [8].

In the prepared copolymer, the degree of unsaturation is determined by the content of itaconic acid repeat units. The molar percentages of itaconic acid are given in Table 1, indicating that PLAX polymers with different degrees of unsaturation were successfully produced with the tested reactors.

Molecular weights of the PLA polymers were measured by SEC, as shown in Table 2. The molecular weight range (Mn) was 5000–16,000 g/mol, aligning with previous studies that indicated that achieving molecular weights above 15,000 g/mol with polycondensation was difficult [8,25]. Molecular weight and Tg values depend on the amount of co-initiator diol used in polymerization. By lowering the diol dosage, PLAX polymers with higher molecular weights are produced. The highest molecular weight polymer produced was P.5 with a diol amount of 1.5 mol%. The low molecular weight polymer P.3 was used for further coating trials, and the targeted properties were reached.

Thermal properties were determined by DSC (Table 2 and Figure S1). The glass transition temperature ranged from −1.4 to 40.8 °C. This indicates a significant variability between batches, showing the ability to tune the polymer properties through their chemical composition, molecular weight, and molecular weight distribution or polydispersity index (PDI). Higher Tg values were obtained for polymers with higher molecular weight (P.2 and P.5), explained by reduced mobility of longer polymer chains. Tg values also depend on the flexibility of monomer units incorporated into the polymer chain. For instance, P.3 contains a higher content of 2,3-butanediol (40 mol%), and it would be expected to have a lower Tg when compared with P.1, P.2, and P.4 (10 and 1.5 mol% 1,4-butanediol). Surprisingly, this was not the case and can be explained by the difference between the flexibility of butanediol molecules used in this study. The P.4 polymerization reaction was conducted at 145 °C, which accounts for the low glass transition temperature.

### 3.2. Scaling up Studies from Lab to Pilot

Comparing reactor types, batches produced with the same recipe in a stirring tank reactor with vertical mixing (Juchheim 2 L) and a vacuum shovel dryer reactor with a horizontal mixer (Lödige 10 L) showed no significant differences in thermal properties (e.g., P.2 and P.5). The glass transition temperature of the polymer produced in the Lödige reactor was marginally higher than that in the Juchheim reactor. This might be due to the better mixing and heat transfer in the Lödige reactor compared to the Juchheim reactor.

A variance in molecular weights can be seen between polymers P.2 and P.5. The average vacuum level in P.2 was maintained at 13 mbar, while in P.5, full vacuum (below 5 mbar) was achieved, potentially contributing to the enhanced molecular weight observed in the P.5 polymer. Overall, these results indicate good processability for polymerization in both reactor types, although it seems that in the Lödige-type reactor, polymers with higher molecular weights and slightly higher glass transition temperatures can be achieved.

### 3.3. Aqueous Dispersions of PLAX Polymers

Three different copolymers were chosen for the dispersion tests based on their molecular weights and glass transition temperatures (Table 2). Two of the polymers, P.4 and P.5, had significantly different Mw (21,360 g/mol and 42,740 g/mol) and Tg (−1.4 °C and 40.8 °C) values, while the P.3 polymer had the mid-range values of Mw and Tg (Mw = 13,390 g/mol, Tg = 10.8 °C).

Dispersions were prepared to investigate the effects of various parameters on dispersion quality, including polymer molecular weight and glass transition temperature, stabilizer grade and dosage, as well as post-treatment. Dispersion quality was evaluated by analyzing the shape and size of the dispersion particles along with basic properties such as pH, conductivity, and viscosity. The initial ratios of PVA and other components were selected based on our previous research [8,25] and subsequently refined to accommodate the specific properties of each polymer through trial and error. All the dispersions were prepared with the same thermomechanical method. The characteristics of the prepared dispersions are presented in Table 3 and Table S2.

### 3.4. Effect of Polymer Molecular Weight and Glass Transition Temperature

To study the effect of the polymer properties—molecular weight and glass transition temperature—a dispersion was prepared from each of the previously mentioned polymers (P.3, P.4, and P.5). Poval grade 40-88 was used as the stabilizer at a 10 wt% concentration relative to the polymer amount in all dispersions.

**Figure 2:**
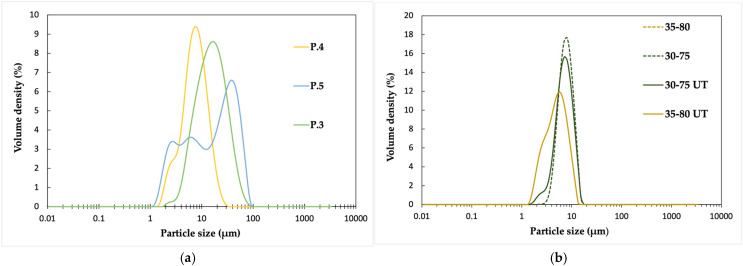
(**a**) Particle size distributions of aqueous dispersions prepared with three different PLAX polymers using Poval 40-88 as dispersion stabilizer. (**b**) Comparison of particle size distributions of PLAX dispersions before and after Ultra Turrax post-treatment using Poval 35-80 and Poval 30-75 dispersion stabilizers.

From Figure 2a, it is apparent that the particle size distributions of D.1 (P.3 polymer) and D.2 (P.4 polymer) are uniform, with the size ranges being between 1, 5 and 30 μm for D.1 and between 2 and 100 μm for D.2. The size distribution of D.3 (P.5 polymer) is bimodal, and the particles have the broadest size range, starting from 1 μm and reaching 100 μm. The microscope images presented in Figure S2 confirm these observations related to the differences between the polymer properties. The size distribution of D.2 is homogeneous, while D.1 and D.3 have particles in several size classes. In all the dispersions, the particles have a spherical shape.

The differences can be explained by polymer properties, including the Mw and Tg. Molecular weight distribution, or polydispersity index (PDI), also has an important influence on the stability of aqueous polymer dispersions. A narrower PDI indicating more similar polymer chain lengths has a positive impact on dispersion stability, reducing the phase separation and sedimentation. In this case, the dispersed particles have a more uniform size distribution and enhanced steric or electrostatic stability, providing more uniform films for coatings and better barrier properties. The more heterogeneous size distribution of D.3 is likely caused by the polymer’s Mn, PDI, and Tg, which are the highest of all three polymers and thus favor the formation of larger particles due to the increased viscosity. Looking purely at the Tg of the polymers, it is obvious that the dispersion D.2 prepared from the polymer with the lowest Tg, P.4, produces the smallest particles and the most uniform size distribution. This is due to the higher mobility of polymer chains during the thermomechanical treatment involved in the preparation of aqueous dispersions. The polymer with a mid-range Tg, P.3, exhibits a uniform main peak, positioned between those of D.2 and D.3 (Figure 2a).

The particle size also seems to reduce with decreasing molecular weight when comparing D.3 to both D.2 and D.1. Polymer P.5, used for D.3 dispersion, has the highest molecular weight and contains longer polymer chains with fewer chain ends and reduced mobility. This affects both the formation of uniform dispersed particles and increased Tg. As can be seen from D.2, the combination of a mid-range Mw and the lowest PDI and Tg values among the compared polymers results in the smallest particle size and most uniform size distribution.

### 3.5. Effect of Dispersion Stabilizer

The effects of five different stabilizer grades with varying degrees of hydrolysis and molecular weight were tested. The dispersions were prepared from the same polymer, P.4. The stabilizer content in all dispersions was 10 wt% of the polymer weight.

**Figure 3:**
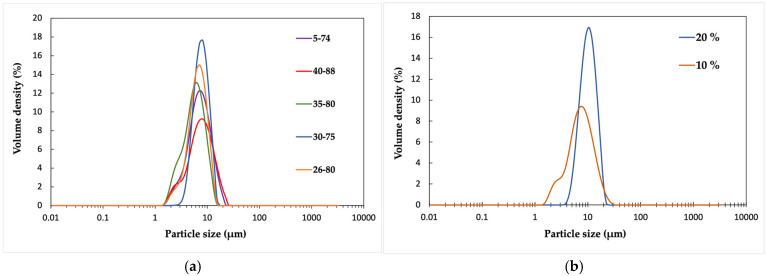
(**a**) Effect of dispersion stabilizer on particle size distribution of PLAX dispersions, and (**b**) comparison of particle size distributions of PLAX dispersions stabilized by Poval 40-88 at different dosages.

In all cases, most of the particles are smaller than 10 μm (Figure 3a). The broadest size range (1.5–25 μm) is produced by the stabilizer Poval 40-88, which has both the highest molecular weight and is the most hydrophilic. Interestingly, the grade with the lowest molecular weight and hydrophilicity, 5-74, has an almost identical particle size distribution. In the dispersions stabilized by the other three grades, 30-75, 35-80, and 26-80, particles of 15–20 μm were observed.

The differences between the influence of stabilizer grades can also be seen in the microscope images (Figure S3). The Poval grades 26-80, 30-75, and 35-80 provided dispersions with similar homogeneous size distributions. The dispersion stabilized by Poval 5-74 has the most heterogeneous size distribution, with the largest particles being up to 30 μm. Although similar in the particle size measurement, the size distribution of Poval 40-88 seems to be slightly less heterogeneous than Poval 5-74 according to the microscope images.

As shown in Table 3, the dispersion prepared with 30-75 exhibited the highest apparent viscosity (632 mPas), which may indicate enhanced stabilizing efficiency and potentially improved film-forming capability.

Compared to commercial aqueous PLA dispersion formulations produced by Miyoshi Oil & Fat Co., Ltd. (Tokyo, Japan), the particle size is in the same range or slightly higher. The particle size in their formulations is 1–5 μm, and the intended use is in packaging and adhesive applications [34].

The effect of the stabilizer dosage on particle size was studied with Poval 40-88 at two concentrations: 10 wt% and 20 wt% of the polymer amount. According to the particle size measurements (Figure 3b), the smallest particles are achieved with the 10 wt% dosage. For the 20 wt% dosage, the size distribution is uniform and ranges between 4 and 20 μm, while for the 10 wt% dosage, the distribution is between 1.5 and 30 μm. The main peak has a shoulder around 3 μm.

### 3.6. Effect of Post-Treatment

Post-treatment with a homogenizer was studied as a method to further reduce the particle size of the dispersions. The effect was demonstrated with two dispersions prepared from the same polymer, P.4, using two different stabilizer grades, Poval 35-80 and Poval 30-75. The dispersions were treated with an Ultra Turrax homogenizer for 15 min at 12,000 rpm.

Post-treatment has a positive effect on the reduction in particle sizes in both dispersions (Figure 2b). In the case of the dispersion prepared with Poval 30-75, the size range broadens due to the new, smaller particles that emerge at around 1.5–3 μm, while the rest of the peak remains the same. This can also be clearly seen in the microscope images (Figure S4), where the number of smaller particles has increased.

In the case of Poval 35-80, the change is different since the main peak has moved slightly to the left and the previously faint shoulder at around 1.5–3 μm is more visible. This means that the largest particles, around 15 μm, have disappeared and that the number of small particles has increased. The change appears quite minor in the particle size results; however, the microscope images (Figure S4) confirmed the disappearance of the large particles.

A higher solids content and thus higher viscosity could potentially increase the shear and therefore promote reduction in the particle size during the homogenizer post-treatment [35]. Here, the dispersions were already diluted to their final solids content before the post-treatment. How performing the homogenizer treatment on an undiluted dispersion affects the particle size distribution remains to be studied. One possible explanation for the weak effect of the post-treatment is that below a certain threshold, the particle size ceases to decrease further. Based on our trial, this threshold would exist close to one micrometer.

### 3.7. Scale-Up of PLAX Dispersion Process

**Figure 4:**
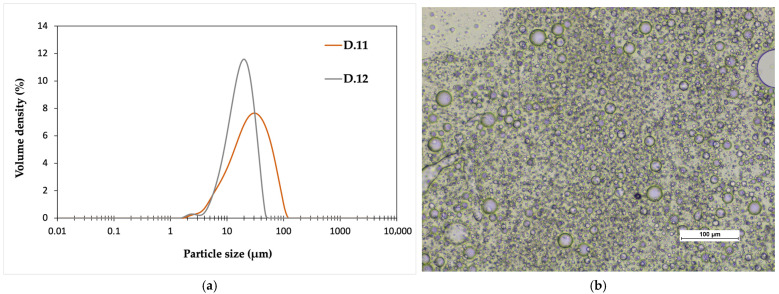
(**a**) Particle size distributions of the upscaled PLAX dispersions and (**b**) microscope image of the D.12 dispersion, 20× magnification.

The dispersion preparation process was scaled up to a 2 L reactor with P.5 polymer. Due to the higher molecular weight of the chosen polymer (Mw= 42,740 g/mol), the dispersion trials were performed under pressure (5–6 barg) at a higher temperature (150 °C). The trials had different mixing speeds: 84 RPM in D.11 and 100 RPM in D.12. In the smaller particle size classes, the particle size distributions of both batches are similar. The largest particles are smaller for D.12, which is most likely due to the increased shear caused by the higher mixing rate, which breaks down the particles (Figure 4a). The same difference in particle size can also be seen in microscope images (Figure 4b).

As can be seen from the scaled-up results, there are several factors contributing to the final properties of the dispersion. As the final product quality has been improved, the variations in the process should be decreased when the process is scaled up to a large scale. The polymer quality and the mixing speed in the dispersion process strongly impact the final dispersion properties. Also, process optimization towards more continuous production would be necessary in future studies before large-scale production.

### 3.8. Coatings and Barrier Characterization

Two PLAX dispersions were selected to be coated onto paper-based substrates using a roll-to-roll semi-pilot SUTCO (surface treatment concept) coater. The formulations of the dispersions are presented in Table 3. The chosen polymers, P.3 and P.4, and the dispersion stabilizer Poval 30-75 produced dispersions with the most uniform size distributions in the earlier dispersion trials. The particle size distributions of the coated dispersions were almost identical (Figure S5). The distributions were uniform, and the size range was between 1.5 and 30 μm.

A metered rod coating method was used for the coating process, and the coating rod applies approximately 28 µm wet coating thickness. Two commercial base papers were used for the coating process. Base paper ‘A’ has a base weight of 65 g/m^2^ and is pre-coated with a barrier layer from the supplier. Base paper ‘B’ has a base weight of 60 g/m^2^ and does not have any pre-coating. The coatings were performed at a speed of 4 m/min, and a combination of infrared and hot-air dryers was used to dry the PLAX dispersion. The coated papers were conditioned at 23 °C and 50% RH for 24 h before their characterization. Table 4 shows the coat weights and water vapor transmission rates (measured according to ASTM E-96 [36]) of the coated samples. The results show that both PLAX dispersions show promising water vapor barriers (Figure S6).

In the earlier trials with similar dispersions, the obtained water vapor transmission rate was 22.8 g/m^2^ with a 10.3 g/m^2^ coat weight [8]. Here, the significantly higher molecular weights of the copolymers (Mn values of 5050 and 10,180 g/mol vs. 2800 g/mol) explain the reduced water vapor passage. Between the two coated dispersions, D.13 and D.14, the dispersion based on the higher molecular weight polymer P.4 (Mn = 10,180 g/mol), D.14, exhibited the lowest WVTR (5.6 g/m^2^/day). Further information on SEM images and barrier properties of these coated samples is available in Nissinen et al. [32].

Also, the lower degree of hydrolysis, i.e., lower hydrophilicity of the dispersion stabilizer (here 75 mol%, previously 88 mol%), contributes to the improved water vapor barrier [8]. The selected dispersion stabilizer grade enabled the formation of uniform dispersions and coatings with good barrier performance, but as only one grade was tested in the coating phase, no direct conclusions can be drawn regarding the influence of stabilizer type on final coating properties.

## 4. Conclusions

In this study, aqueous dispersions were prepared from poly(lactic acid)-based copolymers with a thermomechanical method. The main focus was on the development of PLAX dispersion formulation. The scalability of the process was also demonstrated, and the barrier properties of the coated layers were evaluated.

All the studied effects, excluding the post-treatment, had a major impact on the particle size of the dispersions. The effect of the post-treatment was only minor and could potentially be more intense if the post-treatment was performed on an undiluted dispersion. Between the tested dispersion stabilizer grades, the grades with mid-range viscosity and hydrophilicity values produced the smallest particles, while for the grades with end-range values, the size ranges were broader and almost identical. Further investigation is needed to determine the contribution of each property to particle size. Between the two stabilizer dosages tested, the results are clear: with the 20 wt% dosage, the particle size distribution is more uniform, while the 10 wt% stabilizer dosage leads to the generation of particles in the smaller size classes but also a broader size range. Additional tests with even lower and higher stabilizer dosages would confirm the finding. Further studies should also focus on optimizing the copolymer composition according to the desired dispersion properties. In addition, a detailed evaluation of particles and coatings with suitable analytical methods (e.g., FTIR, SEM, mechanical studies) is needed to provide a comprehensive understanding of the material properties relevant for packaging applications.

With water vapor transmission rates below 10 g/m^2^, the coated dispersions demonstrated excellent barrier properties, highlighting their potential as sustainable alternatives for packaging applications. Notably, the use of higher molecular weight PLAX copolymers further enhanced the barrier performance, indicating a clear correlation between polymer structure and coating functionality.

## Figures and Tables

**Table 1:** The chemical composition of the polymers prepared with the Juchheim 2 L reactor and the Lödige 10 L reactor. Molar percentages are calculated based on the lactic acid amount. LA (lactic acid), IA (itaconic acid), 1,4-BD (1,4-butanediol), and 2,3-BD (2,3-butanediol). The yield is calculated by comparing the amount of produced polymer to the total amount of reagents in the feed.

Sample	Feed Composition, (mol%)				Polymer Composition by ^1^H NMR, (mol%)				Amount (g)	Yield (%)
	LA	IA	1,4-BD	2,3-BD	LA	IA	1,4-BD	2,3-BD		
Polymers prepared with Juchheim 2 L reactor										
P.1	100	10	10	-	100	4.8	10	-	716.0	65.0
P.2	100	10	1.5	-	100	2.7	1.5	-	647.0	63.0
P.3	100	40	-	40	100	12.3	-	40	1077.0	63.0
Polymers prepared with Lödige 10 L reactor										
P.4	100	10	10	-	100	3.6	10	-	2454.0	61.0
P.5	100	10	1.5	-	100	1.0	1.5	-	1869.0	50.0

**Table 2:** Molecular weights (M_n_/M_w)_, polydispersity index (PDI), glass transition temperatures (Tg), and changes in the specific heat capacity (ΔCp) of the PLAX polymers.

Sample	SEC (HFIP)			DSC (2nd Heating)	
	M_n_ (g/mol)	M_w_ (g/mol)	PDI	T_g_ (°C)	ΔCp (J g^−1^ K^−1^)
Polymers prepared with Juchheim 2 L reactor					
P.1	4810	10,990	2.28	10.0	0.59 ± 0.01
P.2	9260	21,480	2.31	38.3	0.52 ± 0.01
P.3	5050	13,390	2.65	10.8	0.57 ± 0.03
Polymers prepared with Lödige 10 L reactor					
P.4	10,180	21,360	2.10	−1.4	0.50 ± 0.01
P.5	15,700	42,740	2.70	40.8	0.55 ± 0.05

**Table 3:** Characteristics of the prepared PLAX dispersions.

Dispersion Sample	Polymer	PVA Grade and Dosage (wt%)	Solids Content (wt%)	pH	Conductivity (mS/cm)	Apparent Viscosity (mPa s) at 50 rpm
Effect of polymer properties						
D.1	P.3	40-88, 10 wt%	33.40	1.99	1.6	173.6
D.2	P.4	40-88, 10 wt%	34.20	2.13	1.5	292.0
D.3	P.5	40-88, 10 wt%	30.80	1.88	1.6	406.0
Effect of PVA stabilizer						
D.4	P.4	5-74, 10 wt%	34.62	1.85	2.0	42.8
D.5	P.4	26-80, 10 wt%	34.11	1.86	1.5	310.0
D.6	P.4	30-75, 10 wt%	33.13	2.04	1.3	632.0
D.7	P.4	35-80, 10 wt%	33.56	1.96	2.1	120.0
D.8	P.4	40-88, 10 wt%	34.20	2.13	1.5	292.0
D.9	P.4	40-88, 10 wt%	34.62	1.85	2.0	42.8
D.10	P.4	40-88, 20 wt%	33.98	2.25	1.2	-
Effect of post-treatment						
D.6	P.4	30-75, 10 wt%	33.13	2.04	1.3	632.0
D.7	P.4	35-80, 10 wt%	33.56	1.96	2.1	120.0
Upscaled dispersions						
D.11	P.5	40-88, 20 wt%	25.98	2.06	3.3	-
D.12	P.5	40-88, 20 wt%	26.57	1.61	3.3	-
Coated dispersions						
D.13	P.3	30-75, 10 wt%	34.92	1.84	1.6	193.6
D.14	P.4	30-75, 10 wt%	-	-	-	-

**Table 4:** Coat weights and water vapor transmission rates of the coated samples.

Property	Base A	A + D.13	A + D.14	Base B	B + D.13	B + D.14
Coat weight (g/m^2^)	-	12.5 ± 0.7	10 ± 0.5	-	11.7 ± 0.2	11.8 ± 0.1
WVTR (23 °C/50% RH) (g/m^2^/day)	14.9 ± 0.7	6.3 ± 1.6	5.6 ± 0.3	20.7 ± 0.7	15.4 ± 1.3	14.9 ± 0.7

## Data Availability

The original contributions presented in this study are included in the article/Supplementary Material. Further inquiries can be directed to the corresponding author.

## Supplementary Materials

The following supporting information can be downloaded at https://www.mdpi.com/article/10.3390/polym17182467/s1: Method Description S1: Characterization of aqueous PLAX dispersions; Method Description S2: Characterization of PLAX dispersion coated samples; Figure S1: DSC thermogram of P.4 polymer; Figure S2: The microscope images of aqueous dispersions prepared with three different PLAX polymers, 20× magnification; Figure S3: Microscope images of PLAX dispersions prepared from same PLAX polymer (P.4) but with different stabilizer grades, 20× magnification; Figure S4: Microscope images of PLAX dispersions before and after post-treatment, 40× magnification; Figure S5: Particle size distributions of the coated PLAX dispersions; Figure S6: Water vapor transmission rates of the coated samples; Table S1: List of tested poly(vinyl alcohol) grades and their properties; Table S2: Volume-weighted particle size distributions as percentiles values for PLAX dispersions prepared by thermomechanical method in presence of poly(vinyl alcohol) dispersion stabilizer. Ref. [37] is cited in Supplementary Materials.

## Abbreviations

The following abbreviations are used in this manuscript:

1,4-BD	1,4-butanediol
2,3-BD	2,3-butanediol
ASTM	American Society for Testing and Materials
DSC	Differential scanning calorimetry
EVA	Ethylene vinyl acetate
HFIP	1,1,1,3,3,3-hexafluoro-2-propanol
IA	Itaconic acid
IR	Infrared
ISO	International organization for standardization
LA	Lactic acid
Mn	Number average molecular weight
Mw	Weight average molecular weight
PDI	Polydispersity index
PLA	Poly(lactic acid)
PLAX	Poly(lactic acid)-based copolymer
PVA	Poly vinyl alcohol
RH	Relative humidity
SEC	Size exclusion chromatography
SUTCO	Surface treatment concept
Tg	Glass transition temperature
Tm	Melting temperature
WVTR	Water vapor transmission rate
