# Supplementary material for: Bio-Based Aqueous Dispersions Based on Unsaturated PLA Polymers for Barrier Packaging Applications
Source: Polymers (Basel). 2025 Sep 12;17(18):2467. doi: 10.3390/polym17182467 (PMC12473213; doi:10.3390/polym17182467)
Supplement: Supplementary file 1 [file polymers-17-02467-s001.zip › polymers-3773444-supplementary.pdf]

Supporting Information

Bio-Based Aqueous Dispersions Based on Unsaturated PLA Polymers for Barrier Packaging Applications

Roosa Hämäläinen, Pauliina Kivinen, Rajesh Koppolu, Eetu Nissinen and Adina Anghelescu-Hakala *

VTT Technical Research Centre of Finland Ltd., P.O. Box 1000, FI-02044 Espoo, Finland; hamalainenroosamaria@gmail.com (R.H.); pauliina.kivinen@vtt.fi (P.K.); rajesh.koppolu@vtt.fi (R.K.); eetu.nissinen@vtt.fi (E.N.)

* Correspondence: adina.anghelescu-hakala@vtt.fi

Method description S1. Characterization of aqueous PLAX dispersions

Visual inspection, solids content, pH, conductivity and viscosity. The fresh dispersions were examined visually to evaluate the consistency and to notice any signs of instability. The solid content of the samples was analyzed directly after preparation with an IR-35 Moisture Analyzer from Denver Instrument (Bohemia, NY, United States).

The other basic properties, pH, conductivity and viscosity, were measured within one day of dispersion preparation. Conductivity was measured with a 4510-conductivity meter from Jenway (Dunmow, United Kingdom) and pH with a 3510-pH meter also from Jenway (Stone, Staff, United Kingdom). Viscosity measurements were performed either with a DV-III ULTRA programmable rheometer from Brookfield (Stoughton, MA, United States) with a spindle providing torque in the optimal range or with an MCR 301 rheometer and a suitable measuring system from Anton Paar (Graz, Austria).

Optical microscopy. Optical microscope images were recorded with a microscope TL19194 from Nikon (Japan) using Measure NIS-Elements software. Magnification ranges of 10X, 20X and 50X were used to image dispersion specimens with transmitted light. The microscope analysis was

performed within one day of the dispersion preparation and the samples were diluted with distilled water with a 1:1 ratio.

Method description S2. Characterization of PLAX dispersion coated samples

Coat weight and thickness. The samples were conditioned in the standard laboratory conditions 23 °C, 50% relative humidity (RH) for 24 hours and cut using a mallet die punch (diameter 74.6 mm, Thwing-Albert Instrument Company; West Berlin, NJ, United States). The coat weight was calculated by substracting the grammage of substrate from the grammages of coated papers. The coating thickness was obtained by comparing the thicknesses of uncoated and coated samples measured with a thickness tester by Lorentzen & Wettre.

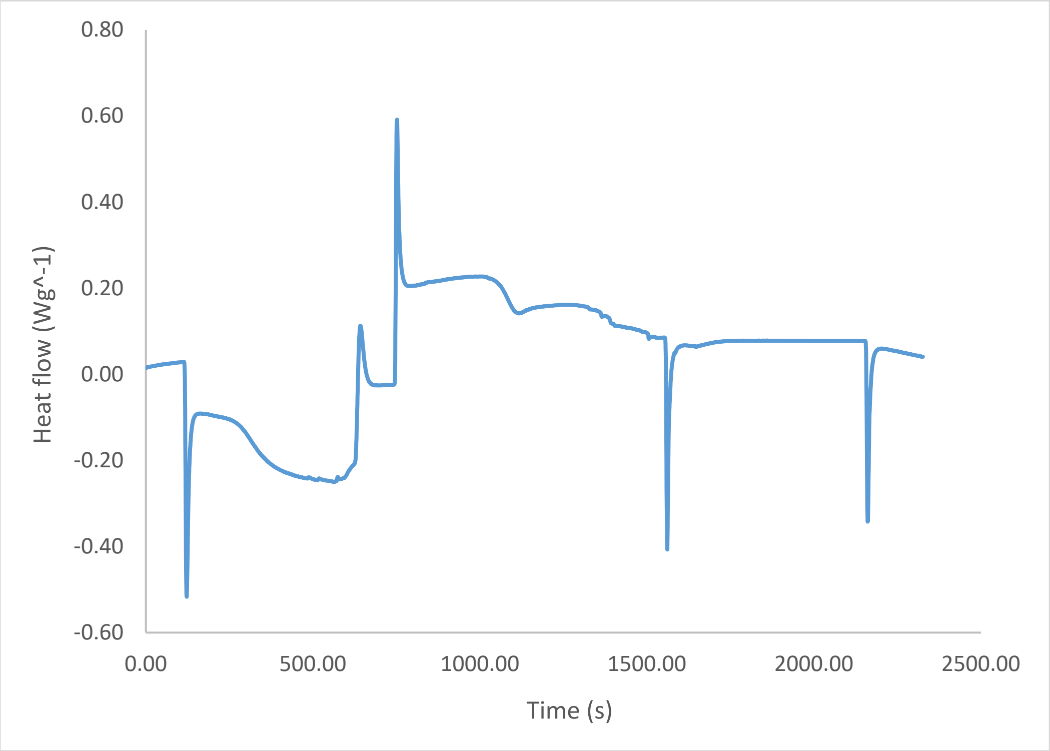

Figure S1. DSC thermogram of P.4 polymer. Heat flow (Wg^{-1}) vs. time (s).

Table S1. List of tested poly(vinyl alcohol) grades and their properties. The pH range is 5.0 - 7.0 for all grades [1].

Grade name		Viscosity (mPas)	Degree of hydrolysis (mol%)
Kuraray Poval™	5-74	4.2 - 5.0	72.5 - 73.5
	26-80	21.0 - 31.0	78.0 - 81.0
	30-75	28.0 - 32.0	74.0 - 76.0
	35-80	32.0 - 38.0	79.0 - 81.0
	40-88	38.0 - 42.0	87.0 - 89.0

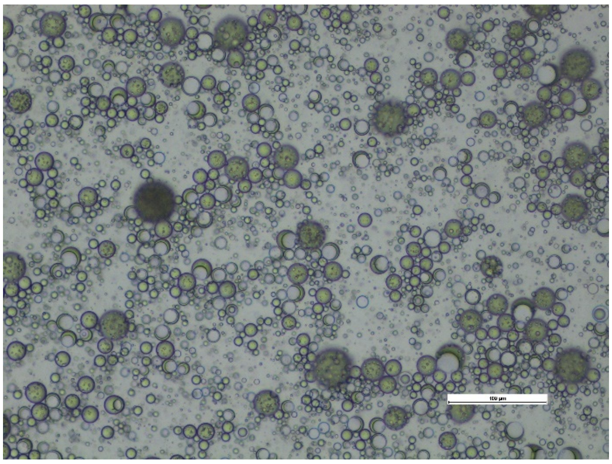

P.3 (D.1)

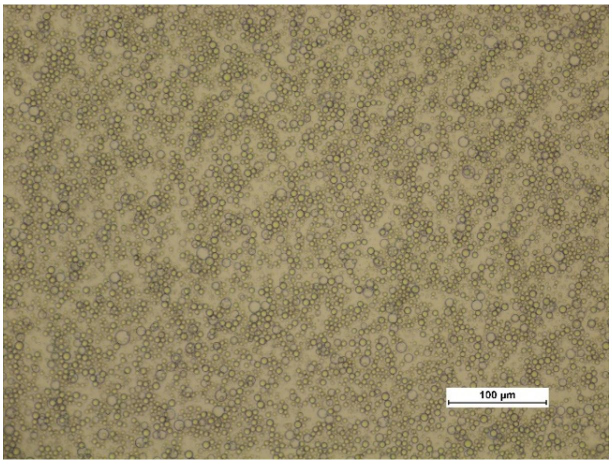

P.4 (D.2)

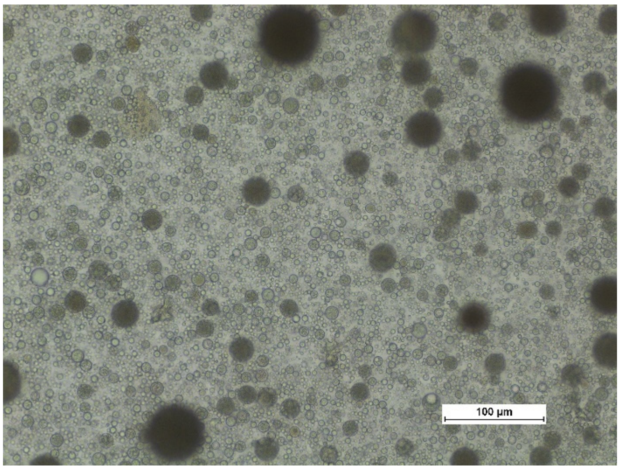

P.5 (D.3)

Figure S2. The microscope images of aqueous dispersions prepared with three different PLAX polymers, 20X magnification.

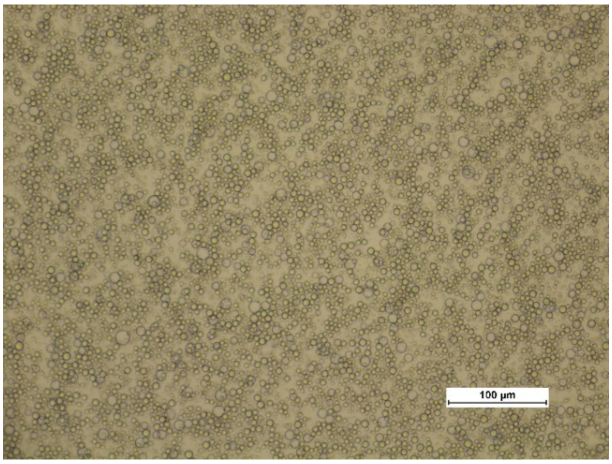

Poval 40-88 (D.8)

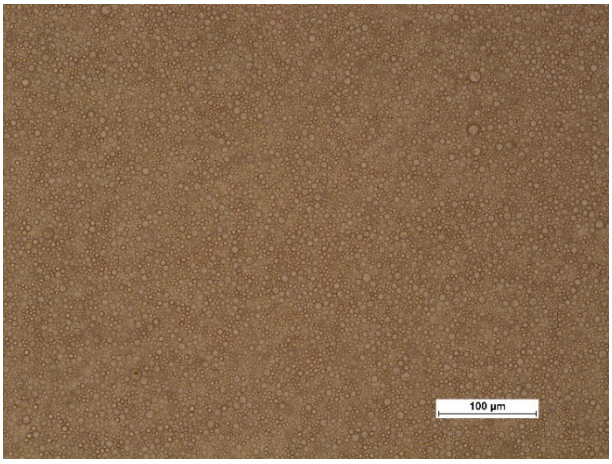

Poval 26-80 (D.5)

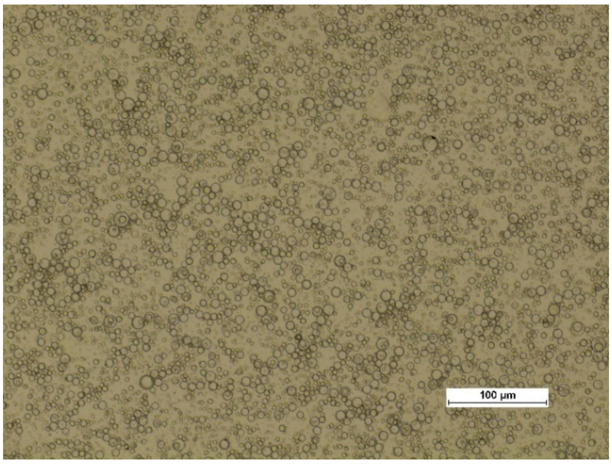

Poval 30-75 (D.6)

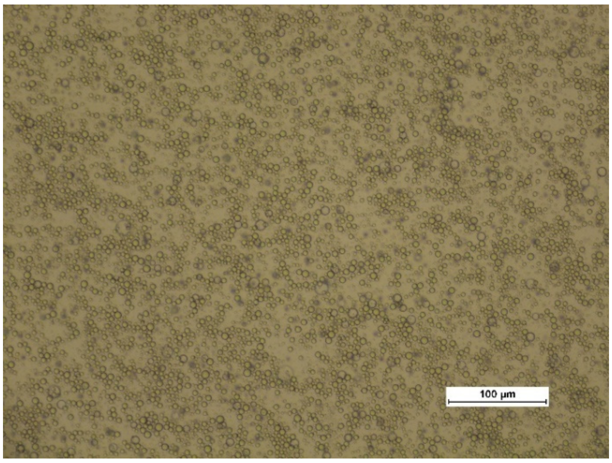

Poval 35-80 (D.7)

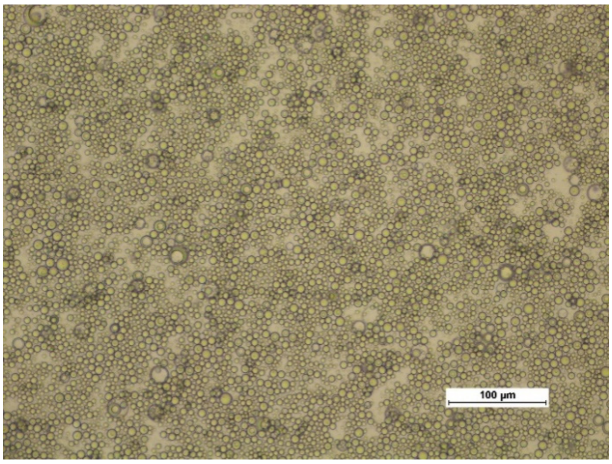

Poval 5-74 (D.4)

Figure S3. Microscope images of PLAX dispersions prepared from same PLAX polymer (P.4) but with different stabilizer grades, 20X magnification.

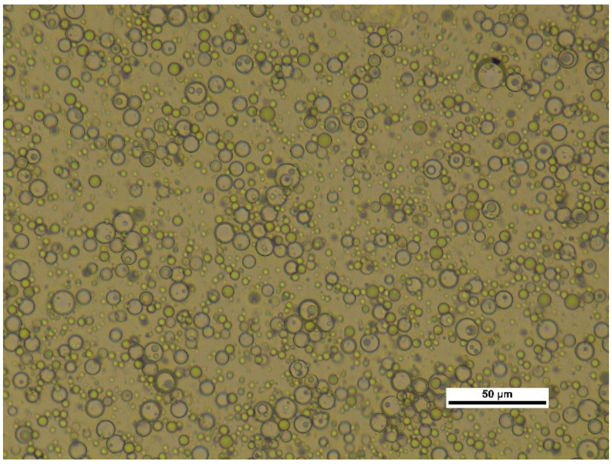

Poval 30-75 (D.6)

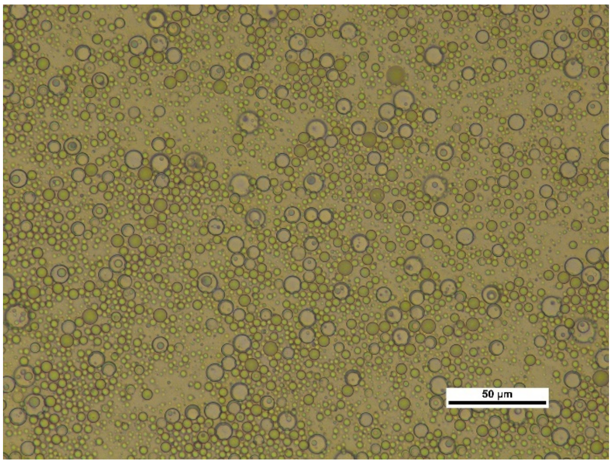

Poval 30-75 UT

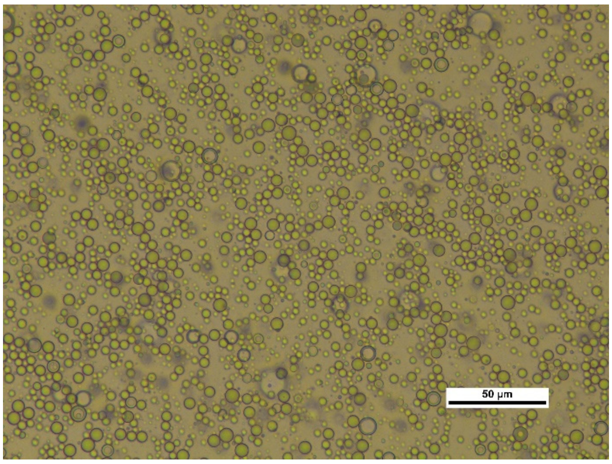

Poval 35-80 (D.7)

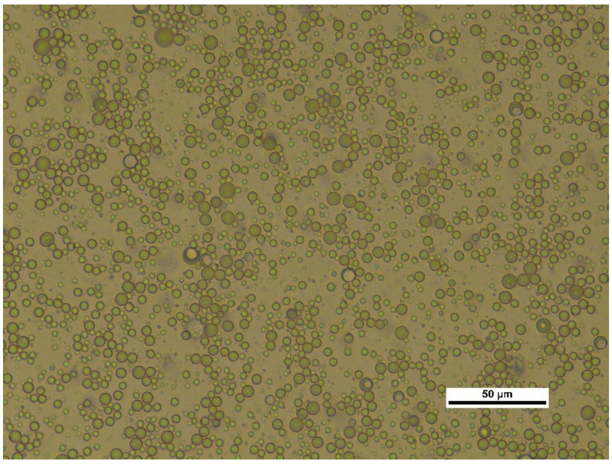

Poval 35-80 UT

Figure S4. Microscope images of PLAX dispersions before and after post-treatment, 40X magnification. The dispersions were prepared from the same polymer P.4. UT = after the Ultra Turrax post-treatment.

Table S2. Volume-weighted particle size distributions as percentiles values for PLAX dispersions prepared by thermomechanical method in presence of poly(vinyl alcohol) dispersion stabilizer.

Sample	Dv (10) (µm)	Dv (50) (µm)	Dv (90) (µm)
**Effect of polymer molecular weight and glass transition temperature**			
D.1	6.35	15.4	35.0
D.2	3.19	7.38	14.3
D.3	2.66	17.2	55.0
**Effect of stabilizer grade**			
D.4	3.36	6.97	12.8
D.5	3.59	6.59	10.5
D.6	5.04	7.53	11.0
D.7	2.80	5.66	9.45
D.8	3.19	7.38	14.3
**Effect of stabilizer dosage**			
D.9	3.36	6.97	12.8
D.10	6.33	9.97	15.2
**Effect of post-treatment**			
D.6	5.04	7.53	11.0
D.6 post-treated	4.04	7.01	11.2
D.7	2.80	5.66	9.45
D.7 post-treated	2.50	5.18	8.90
**Upscaled dispersions**			
D.11	8.15	23.6	61.4
D.12	9.33	26.4	219
**Coated dispersions**			
D.13	5.73	10.5	18.4
D.14	6.22	11.4	20.7

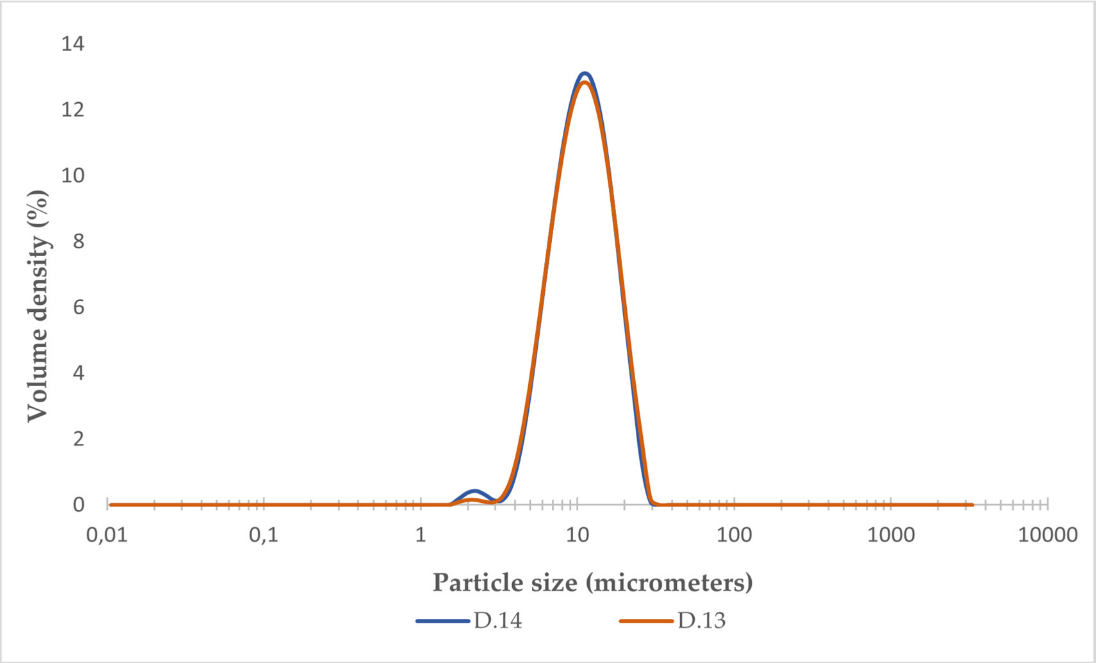

Figure S5. Particle size distributions of the coated PLAX dispersions.

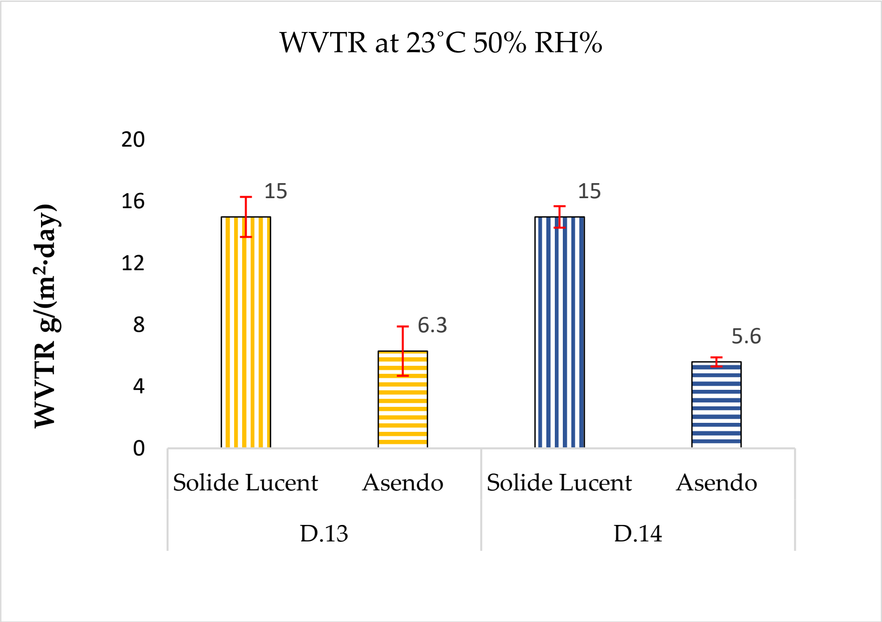

Figure S6. Water vapor transmission rates of the coated samples.

References

1. Kuraray PovalTM, ExcevalTM & ElvanolTM Resins Technical Data Sheet. Available online: https://www.kuraray-poval.com/fileadmin/user_upload/KURARAY_POVAL/technical_information/grades_by_region/grades_poval/TDS-KURARAY_POVAL_TM_-EXCEVAL_TM_-ELVANOL_TM-ENGLISH.pdf (accessed on 13 June 2025).
